# Corrosion Behavior of X80 Steel with Coupled Coating Defects under Alternating Current Interference in Alkaline Environment

**DOI:** 10.3390/ma10070720

**Published:** 2017-06-28

**Authors:** Zhong Li, Caiyu Li, Hongchang Qian, Jun Li, Liang Huang, Cuiwei Du

**Affiliations:** 1 Department of Chemical and Biomolecular Engineering, Institute for Corrosion and Multiphase Technology, Ohio University, Athens, OH 45701, USA; zl129616@ohio.edu; 2 Corrosion and Protection Center, University of Science and Technology Beijing, Beijing 100083, China; caiyuli567@163.com (C.L.); qhcgcc@163.com (H.Q.); B20160524@xs.ustb.edu.cn (J.L.); huangliang063@126.com (L.H.)

**Keywords:** corrosion, X80 steel, coating defect, alternating current interference

## Abstract

The corrosion behavior of X80 steel in the presence of coupled coating defects was simulated and studied under the interference of alternating current (AC) in an alkaline environment. The results from electrochemical measurements showed that the electrode potential of the coating defect with the smaller exposed area was lower than that with the larger area, which indicated that the steel with the smaller coating defect was more prone to corrosion. The result of weight loss tests also showed that the smaller coating defect had induced a higher corrosion rate. However, the corrosion rate of X80 steel at the larger coating defect decreased gradually with the increase of the larger defect area at a constant smaller defect area. The corrosion morphology images showed that the coating defects with smaller areas suffered from more severe pitting corrosion.

## 1. Introduction

Given the increasing demand for energy, electric power and electrified railways, many pipelines are being buried in parallel or are intersecting with feeder cables and subway rails [1,2,3]. This pipeline configuration is significantly distinct from the alternating current (AC)-induced corrosion of pipelines [4,5,6,7]. AC interference, induced by magnetic field from feeder cables or leakage current from power supply systems, is a hotspot in international corrosion research. Early studies indicated that AC can cause pitting corrosion and enhance stress corrosion cracking (SCC) sensitivity [8,9,10,11,12,13,14,15]. These factors can shorten the life of pipeline steels, thus leading to oil and gas blowout accidents, environmental pollution, and casualties. Therefore, research on the mechanism of metal corrosion affected by AC and the relevant corrosion mitigation methods are significantly important.

The AC-induced corrosion of steel [16,17,18] has been extensively studied. AC interference can cause the severe corrosion of metal materials. However, the mechanism of corrosion under AC interference is still unclear. For example, Wang et al. [14] presented that the corrosion potential of X80 and X100 steels could be shifted with AC amplitude and frequency. The anodic process was under the charge-transfer control, and the anodic Tafel slopes increased with increasing AC magnitudes. The controlling factors of cathodic processes changed with the AC amplitude. Lalvani et al. [19,20,21] adopted a model with a similar solution and concluded that the corrosion current was correlated with the absolute ratio of the anodic and cathodic Tafel slopes. Furthermore, the corrosion rate was generally determined by the direct current (DC) potential maintained at the anode. Zhang et al. [22] developed a mathematical model and found that the AC-induced corrosion rate was independent of the corrosion current in the absence of alternating voltage. They also found that the AC-induced corrosion rate increased with the peak potentials of the AC signal. Ono et al. [23] proposed that a correlation existed between the half-cycle on AC and the corrosion of the aluminum etch process. The cathodic half-cycle enhanced the passivation of pits developed during the preceding anodic half-cycle, thus making pit nucleation random. An anodic pulse without cathodic half-cycle produced defects because of the preferential pit nucleation on pits produced in the preceding anodic half-cycle. Xu et al. [24,25] studied the effects of AC on the performance of cathodic protection (CP) on a 16 Mn pipeline steel in a simulated soil solution. The presence of AC interference decreased the effectiveness of CP in protecting steel from corrosion.

During transportation and use, the improper machining, uneven painting, and blistering will inevitably cause some polymeric coating defects of varying sizes on pipeline surfaces. AC interference penetrates these defects via the buried pipeline overhead electric system and power supply equipment. However, few studies have been conducted on the corrosion behavior of buried pipelines with different sizes of coating defects under AC interference in simulated soil solutions. Fu et al. [26] reported that an X65 steel electrode containing a 1 mm coated defect has higher electrochemical dissolution activity than an electrode containing a 10 mm defect on the AC-induced corrosion. Nevertheless, their investigation focuses on the AC-induced corrosion within single coating defects on pipeline steel and excludes the study of interconnected coating defects.

The aim of this paper is to understand the effects of AC interference on the corrosion behaviors of buried X80 pipeline steel in the presence of coupled coating defects, via a combination of electrochemical tests and weight loss tests in a simulated soil solution. Laboratory experiments were conducted by using a self-designed circuit connecting two X80 steel specimens with different exposed areas. The corrosion potentials, AC/DC currents and corrosion rates of these two specimens were measured and analyzed in detail to provide an accurate explanation for the corrosion behaviors of X80 steel in the presence of coupled coating defects under the influence of AC interference. The corresponding corrosion morphologies of X80 steel at the coating defects were also investigated.

## 2. Experimental

### 2.1. Electrode and Solution

**Figure 1:**
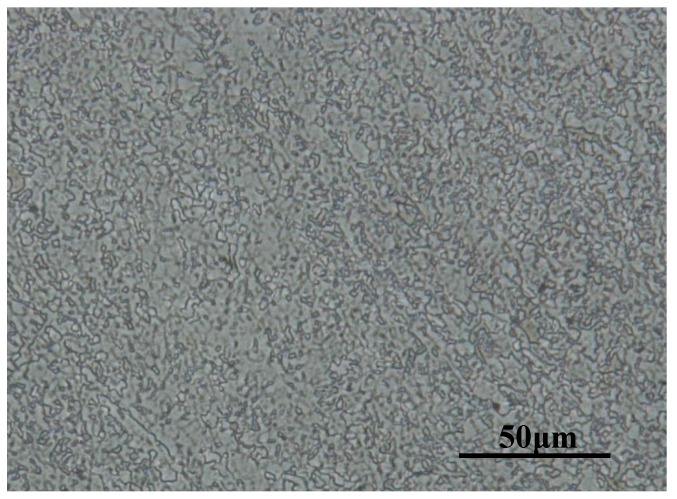
The microstructure of X80 pipeline steel.

The test specimens were cut from a hot-rolled plate of X80 pipeline steel. The composition of the steel was as follows (wt %): C, 0.061; Si, 0.19; Mn, 1.75; P, 0.012; S, 0.001; Cr, 0.033; Ni, 0.21; Cu, 0.16; Nb, 0.045; and Fe balance. The microstructure of the steel was observed by optical microscope (VHX-2000, KEYENCE, Tokyo, Japan) and is shown in Figure 1. X80 pipeline steel was composed of a large amount of ferrite and a small amount of granular bainite. The distributions of the two microstructures were uniform.

China’s northwest Xinjiang region is an important hub of buried pipelines for oil and gas transmission. The soil in this area is typical alkaline soil with high salinity, low humidity, and good air permeability. The experimental simulated solution was prepared based on soil composition from the Korla region in Xinjiang. The chemical compositions of the simulated solution are shown in Table 1. 

### 2.2. Electrochemical Tests

The X80 steel were machined into three dimensions of 10 mm × 10 mm × 3 mm, 20 mm × 20 mm × 3 mm and 30 mm × 30 mm × 3 mm and then coated with epoxy resin leaving the exposed surface areas of 1, 4, and 9 cm^2^, respectively, as working electrodes (WEs). These WEs were coupled into three pairs in accordance to the ratio of the exposed area of WE 1 to that of WE 2:

Pair 1, 10 × 10/10 × 10 (mm^2^);
Pair 2, 10 × 10/20 × 20 (mm^2^);
Pair 3, 10 × 10/30 × 30 (mm^2^).

The open circuit potentials, corrosion products, and corrosion rates of every pair were investigated.

**Figure 2:**
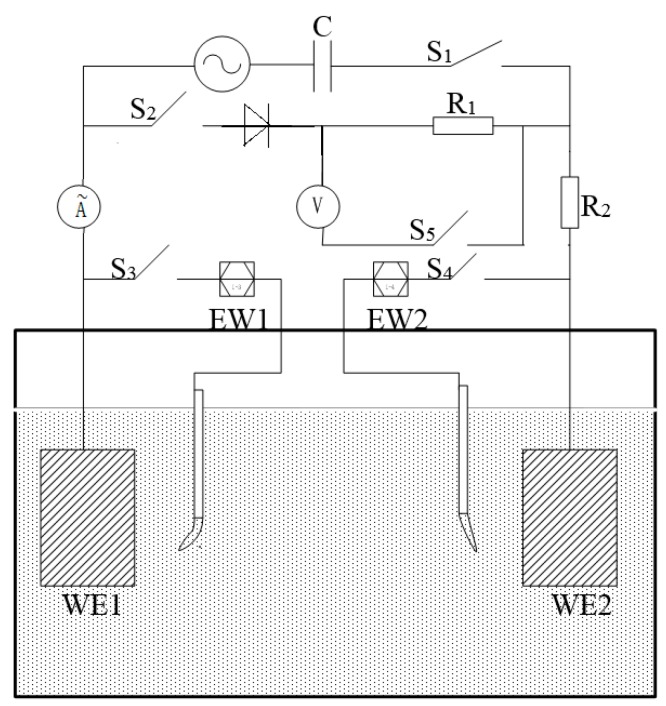
The experimental set-up configuration of electrochemical test.

Each pair had four parallel samples. The working face of the electrode was ground sequentially to 1500 grit emery paper and then successively cleaned using acetone and alcohol, followed by air drying. Real-time experiment electrode potentials were tested by an electrochemical workstation (CorrTest CS300, Wuhan, China). In this work, two specimens with different exposed areas (WE1 and WE2) were connected with an electric circuit to simulate the coupled effect of two nearby coating defects on the same pipeline surface, as shown in Figure 2. Two saturated calomel electrodes with basically the same potentials were used as the reference electrodes. The digital display scope function signal generator was used to apply 20 Vpp AC at 50 Hz on WE1 and WE2. The capacitance in Figure 2 was used to prevent the AC interference to the electrochemical test system. R_1_ was applied to simulate soil and pipe resistance. R_2_ was designed to detect the current of the circuit through measuring the potential drop of R_2_. The values of R_2_ and R_1_ were 50 and 100 Ω, respectively. An Owan oscilloscope was used to monitor the potential fluctuations of R_2_ in real time. S_1_, S_2_, S_3_, S_4_, and S_5_ were used to control the circuit. All tests were performed at room temperature (25 °C).

After the test, the specimens were cleaned by deionized water and dipped into 50% HCl solution with hexamethylenetetramine added in for 10 minutes to remove the corrosion products. The specimens were then weighed after cleaning by deionized water and absolute ethyl alcohol. Thereafter, the specimens were air dried. The corrosion rates were calculated by Equation (1):

(1) $$\nu =\frac{\Delta W}{S\times T}$$

Δ*W*—the loss weight of specimen weight (g);

*S*—specimens area (cm^2^);

*T*—time (day).

The micromorphology and microstructure of the specimen surface were observed by Quanta 250 scanning electron microscopy (SEM, FEI Quanta 250, Hillsboro, OR, USA) and energy dispersive spectroscopy (EDS, FEI Quanta 250, Hillsboro, OR, USA).

### 2.3. Electrochemical Parameter Measurement

To simulate the effect of AC interference on the potential of each sample, the different area ratios of the WE1-WE2 was investigated with and without AC interference, as shown in Figure 2. The measurement was performed in four steps as follows:

Step 1: Switch S_1_/S_5_ were all turned off and S_2_/S_3_/S_4_ turned on to measure the open circuit potential of the WE1–WE2 without alternating current interference.

Step 2: When the open circuit potential was stable, the S_1_ and S_5_ were turned on to establish the AC interference between the two specimens.

Step 3: Switch S_1_/S_2_ was turned off. The WE1 and WE2 were not connected to each other. WE1 and WE2 were in free corrosion.

Step 4: Switch S_2_ was turned on. The experiment was restored to Step 1.

The test time of every step was 2000 s. 

In order to monitor the DC and AC current components flowing between two coupled specimens under AC interference, S_3_ and S_4_ were turned off but other switches were turned on to avoid the interference from the electrochemical workstation to the oscilloscope. The monitoring time was 7 d. In the measurement, the oscilloscope was set to a mode that can monitor the DC and AC components of potential signal simultaneously. The measured potential signals were then converted into current signals.

## 3. Results and Discussion

### 3.1. Electrochemical Analyses

**Figure 3:**
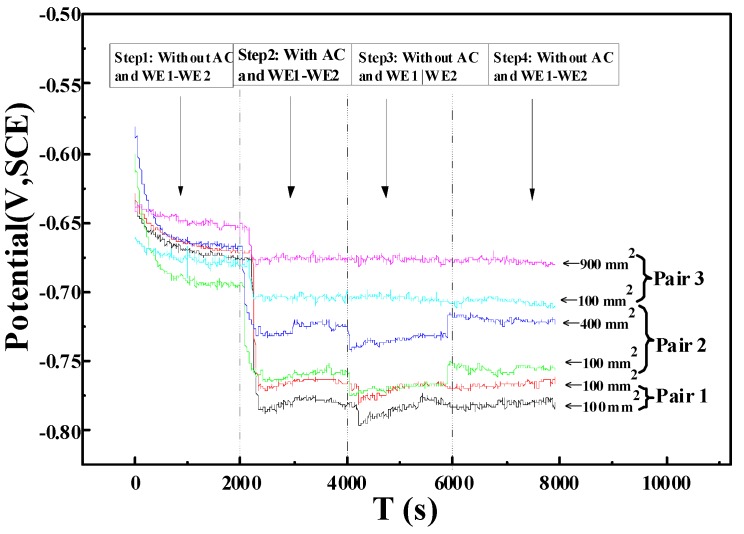
The open circuit potentials of WE1 and WE2 with different area ratios under alternating current (AC) interference.

To evaluate the influence of different area ratios on the corrosion tendency of X80 steel at the coating defect under AC interference, the open circuit potentials of WE1 and WE2 in different steps were measured and shown in Figure 3. In each experiment, the electrode potential on the specimen with a smaller exposed area was lower, thus indicating that the specimen was more likely to corrode than the larger specimen. In terms of the four steps of electrode potential, the potentials in the second and fourth steps were the same. However, the electrode potential jumps for pair 3 are small when experimental conditions were changed which proves the existence of electric current in the experiment circuit. Compared with the specimens with larger exposed areas, the specimen with a smaller area obviously exhibits a higher tendency of corrosion. Upon the same AC current application, the surface current density of the specimen with a smaller exposed area was higher than those with larger areas. Therefore, under the same solution resistance, the electrode potential of the specimen of the smaller area was more negative than that of the larger area.

**Figure 4:**
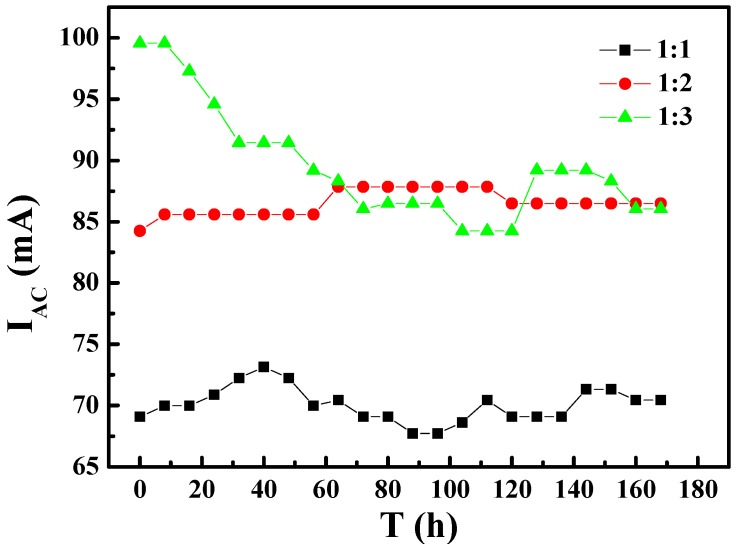
The variation of AC currents on the specimens of different area ratios with immersion time: (1) area ratio of 1:1; (2) area ratio of 1:2; (3) area ratio of 1:3.

**Figure 5:**
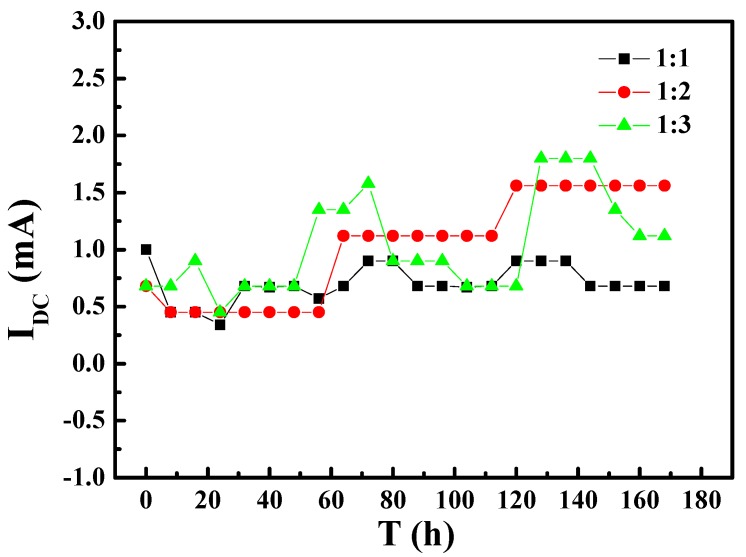
The variation of direct current (DC) under different area ratios with immersion time: (1) area ratio of 1:1; (2) area ratio of 1:2; (3) area ratio of 1:3.

To better understand the influence of AC interference on the corrosion tendency of the specimens with different area ratios, the AC and DC currents in the test circuit were measured. Figure 4 and Figure 5 show the variation of AC and DC currents on the specimens of different area ratios with immersion time, respectively. As shown in Figure 4, when the exposed areas of WE1 and WE2 are different, the AC current signal was higher than that for ratio 1:1. The AC current was highest for ratio 1:3 at first and decreased to the level for ratio 1:2 after the specimens had immersed for about 65 h. Figure 5 shows no obvious relationship between the magnitude of the DC current and area ratio of WE1 to WE2, and the DC value fluctuated within a range of approximately 1 ± 1 mA. These results suggested that part of the AC current was converted into a DC current, whereas the rest was used to promote the anodic reaction and other reactions.

### 3.2. Corrosion Morphology

**Figure 6:**
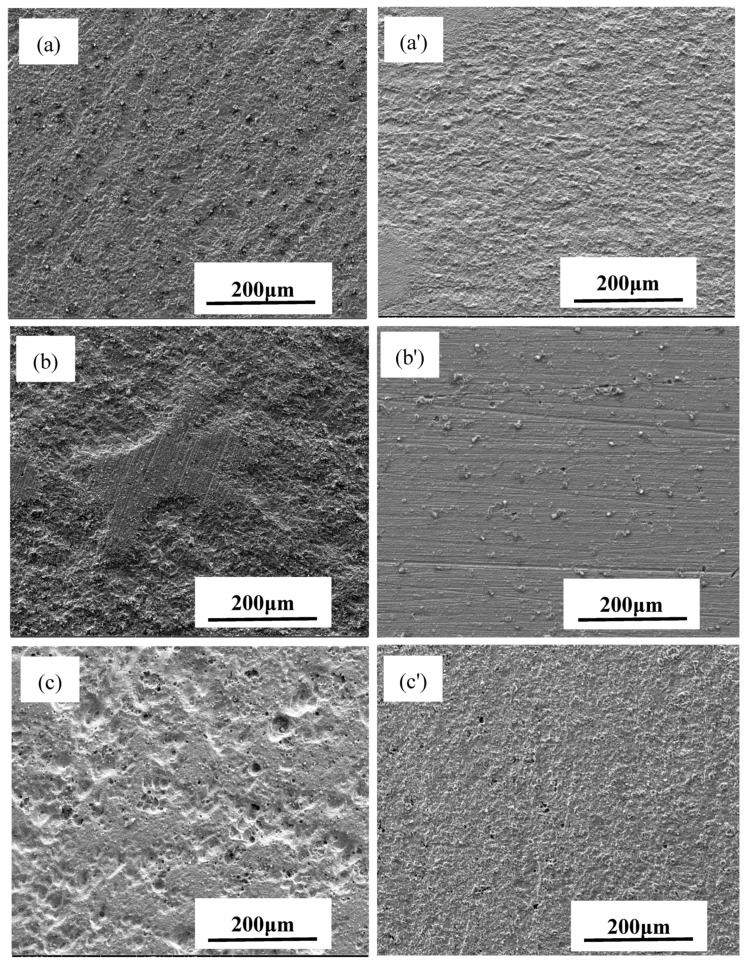
Corrosion morphology of WE1 (n) and WE2 (n’) at different area ratio: (**a**) 1:1; (**b**) 1:2; (**c**) 1:3 under AC interference for seven days after removal of corrosion products.

After the corrosion products were removed, the morphologies of the tested WE1 and WE2 were observed (Figure 6). When the surface areas of WE1 and WE2 were the same, the WE1 and WE2 exhibited uniform corrosion with similar corrosion degrees, and pitting corrosion was not observed. With the increasing area ratio of WE1 to WE2, the WE1 surfaces exhibited the characteristics of non-uniform corrosion, and the density and depth of the corrosion pits increased gradually with the increasing area ratio of WE1 and WE2. When the area ratio of WE1 and WE2 was largest, the surface of the WE1 showed numerous disc-like corrosion pits and a trend of pit integration was observed. In contrast, the WE2 surfaces always showed the feature of uniform corrosion. With increasing WE1 to WE2 area ratio, the current density on the surface of the WE1 became larger than that of WE2, and the potential difference became larger. Thus, the WE1 was most susceptible to corrosion when the area ratio was highest. Beyond that, Xinjiang soil is known for the high concentration of Cl^-^ ions, which can destroy the surface passivation film and promote the occurrence of pitting. Thus, a greater number of deeper pits were observed on the WE1 surface [1].

### 3.3. Corrosion Rates

**Figure 7:**
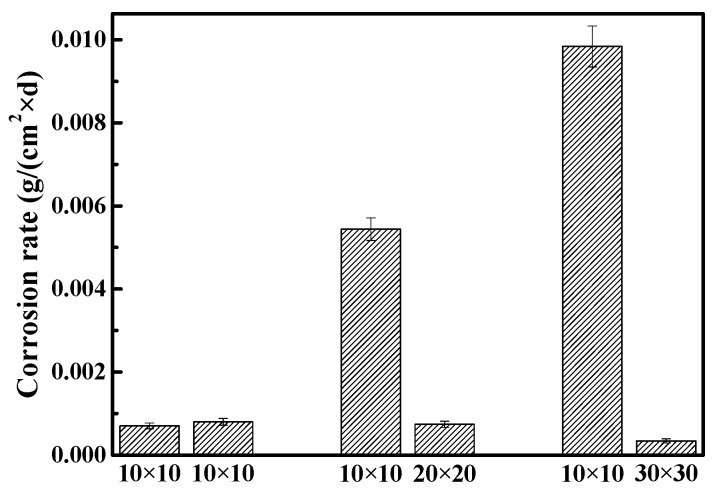
Corrosion rates of WE1 and WE2 with different area ratios under AC interference.

The effect of the difference in the WE1 to WE2 area ratio on the corrosion rate was studied through weight loss measurements. As shown in Figure 7, the corrosion rate of WE1 was at a similar level to that of WE2 when the exposed areas of WE1 and WE2 were the same. With the increasing area ratio of WE1 to WE2, the corrosion rate of WE1 increased rapidly while the corrosion rate of the WE2 gradually decreased. When the area ratio of the group was 1:3, the corrosion rate of WE1 increased to ~ 30 times as much as that of WE2. This result indicated that the specimen with a smaller exposed area will suffer more serious corrosion with the increased area ratio.

### 3.4. EDS Analysis of the Corrosion Products

**Figure 8:**
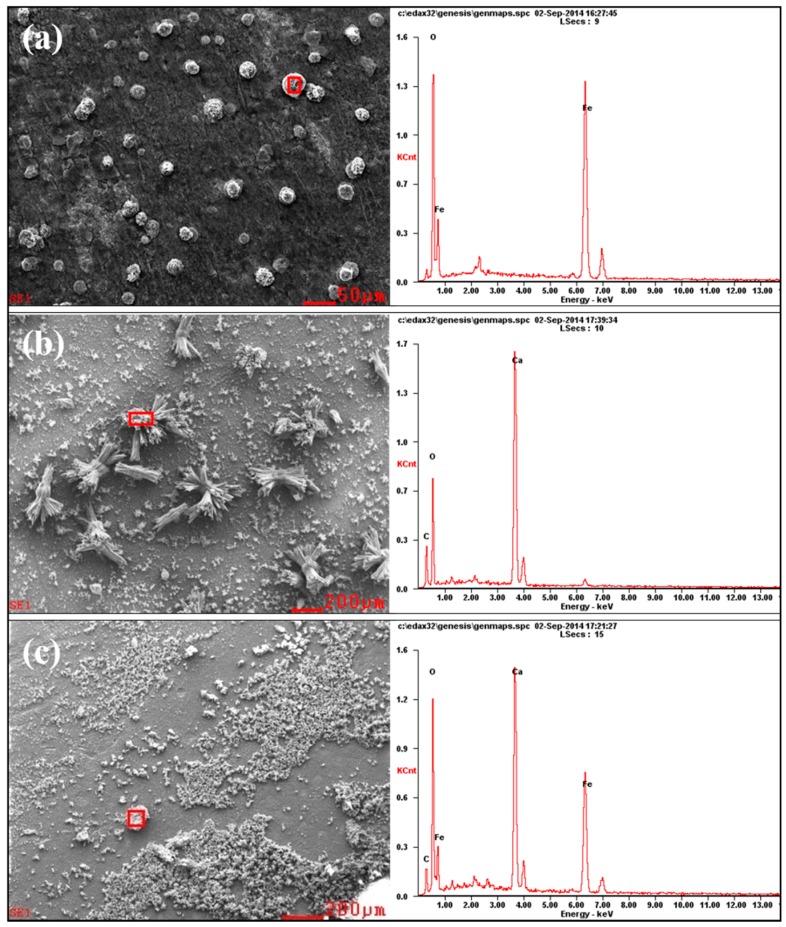
SEM and energy dispersive spectroscopy (EDS) analysis of corrosion products of WE2 with different area ratios of WE2: (**a**) 1:1; (**b**) 1:2; (**c**) 1:3 under AC interference.

In order to provide insights on the electrochemical reactions of the electrode surfaces under different area ratios, the inner layer of the corrosion product on different WE2 surfaces was analyzed by EDS (Figure 8). Iron oxides can be observed on WE2 when the area ratio between WE1 and WE2 was 1:1 (Figure 8a), while calcium salts (e.g., calcium carbonate) have appeared when the area ratios were 1:2 (Figure 8b) and 1:3 (Figure 8c). It can be inferred that the increased area of WE2 can promote the cathodic processes on WE2, which can lead to the increased pH and the precipitation of calcium salts under the rust layer. In the case of 1:1, pH may not increase enough to allow calcium salts to precipitate. Meanwhile, the hydrolysis of ferrous ion would acidify the solution which can hinder the precipitation of calcium salts, too. At the same time, it can be seen from Figure 5 and Figure 6 that the anode processes were promoted under the above conditions. It can be further perceived that the AC interference had promoted the cathodic other than anodic reactions on WE2, and meanwhile accelerate more anodic reactions on the adjacent coating defect (WE1). This is one of the mean reasons why WE1 has a higher corrosion rate with the increased area of WE2. Additionally, an increase in the area of WE2 means that the equivalent resistance of the circuit was reduced. Therefore, under the same AC potential, the AC current density increases with the increased area of WE2, which enhances the cathodic and anodic processes on the electrode surface. The above findings were in good agreement with the results shown in Figure 3, Figure 4, Figure 5, Figure 6 and Figure 7.

In addition, as the area of WE2 increases, the corrosion mode of WE1 greatly changes from general corrosion to pitting, which both increases the effective area of WE1 and makes it easier to form a lower pH environment in pits at the same time (Figure 6) while further promoting the processes of corrosion. For all reasons above, the anodic and cathodic processes were promoted simultaneously. This promotion will be increased with the exposed time. Figure 5 and Figure 6 are consistent with the above discussion. The effect of these factors is significant; a 20% increase in AC current leads to a difference in corrosions rates of WE1 by as much as a factor of 10.

## 4. Conclusions

In this work, the effect of a coupled coating defects on the corrosion potential and corrosion rate of X80 pipeline steel at the coating defects was studied under AC interference, and the following conclusions were obtained:

(1) The open circuit potential of the X80 steel specimen with a smaller coating defect area was lower than those with larger coating defects. This result showed that the specimen with the smaller coating defect was more prone to corrosion.
(2) The AC current induced by the AC interference on X80 steel with different coating defect areas increases with the same area of WE1 and increasing area of WE2. The DC signal was also induced between the two coating defects under AC interference.
(3) Under the action of AC/DC currents, the corrosion rate of the X80 steel at the smaller coating defect increased significantly and that of X80 steel at the larger defect decreased gradually with the increase of the larger defect area at constant smaller defect area.

## Figures and Tables

**Table 1:** Chemical compositions of the simulated solution (g·L^−1^).

pH	NaCl	Na_2_SO_4_	NaHCO_3_	KNO_3_	MgCl_2_·6H_2_O	CaCl_2_
9.4	3.4945	1.2603	0.146	0.2152	0.3383	0.1221
